# Regional Magnetic Resonance Imaging Measures for Multivariate Analysis in Alzheimer’s Disease and Mild Cognitive Impairment

**DOI:** 10.1007/s10548-012-0246-x

**Published:** 2012-08-14

**Authors:** Eric Westman, Carlos Aguilar, J-Sebastian Muehlboeck, Andrew Simmons

**Affiliations:** 1 Department of Neuroimaging, Institute of Psychiatry, King’s College London, De Crespigny Park, London, SE5 8AF UK; 2 Department of Neurobiology, Care Sciences and Society, Karolinska Institutet, Stockholm, Sweden; 3 NIHR Biomedical Research Centre for Mental Health, London, UK

**Keywords:** Freesurfer, MRI, OPLS, AD, MCI conversion, Sensitivity, Specificity

## Abstract

Automated structural magnetic resonance imaging (MRI) processing pipelines are gaining popularity for Alzheimer’s disease (AD) research. They generate regional volumes, cortical thickness measures and other measures, which can be used as input for multivariate analysis. It is not clear which combination of measures and normalization approach are most useful for AD classification and to predict mild cognitive impairment (MCI) conversion. The current study includes MRI scans from 699 subjects [AD, MCI and controls (CTL)] from the Alzheimer’s disease Neuroimaging Initiative (ADNI). The Freesurfer pipeline was used to generate regional volume, cortical thickness, gray matter volume, surface area, mean curvature, gaussian curvature, folding index and curvature index measures. 259 variables were used for orthogonal partial least square to latent structures (OPLS) multivariate analysis. Normalisation approaches were explored and the optimal combination of measures determined. Results indicate that cortical thickness measures should not be normalized, while volumes should probably be normalized by intracranial volume (ICV). Combining regional cortical thickness measures (not normalized) with cortical and subcortical volumes (normalized with ICV) using OPLS gave a prediction accuracy of 91.5 % when distinguishing AD versus CTL. This model prospectively predicted future decline from MCI to AD with 75.9 % of converters correctly classified. Normalization strategy did not have a significant effect on the accuracies of multivariate models containing multiple MRI measures for this large dataset. The appropriate choice of input for multivariate analysis in AD and MCI is of great importance. The results support the use of un-normalised cortical thickness measures and volumes normalised by ICV.

## Introduction

Alzheimer’s disease (AD) is the most common form of dementia in the ageing population of today. The estimated cost of dementia worldwide has been calculated as 315.4 billion USD based on an estimated 29.3 million demented patients in 2005 (Wimo et al. 2007). The number of patients with AD has been predicted to quadruple by 2050 (Brookmeyer et al. 2007). The disease is characterized by a gradual loss of cognitive functions, such as episodic memory. The two major pathological hallmarks of AD are extracellular plaques and intracellular tangles. Plaques and tangles are built of aggregates of Aβ (Glenner and Wong 1984; Masters et al. 1985) and hyperphosphorylated tau (Goedert et al. 1991), respectively. Other characteristics of AD are synaptic loss and neuronal cell death, leading to brain atrophy. Magnetic resonance imaging (MRI) provides structural information about the brain and has for many years been widely used for early detection and diagnosis of AD (O’Brien 2007; Ries et al. 2008). The way in which AD atrophy progresses through the brain has been described by Braak and Braak (1991). Atrophy typically starts in the medial temporal and limbic areas, subsequently spreading to parietal association areas and finally to frontal and primary cortices. For many years studies have focused on single structures in the medial temporal lobe for the early diagnosis of AD, such as hippocampus and entorhinal cortex (Fox et al. 1996; Jack et al. 1992, 1997; Juottonen et al. 1999). In recent years however, research has focused on combining different regions to look at patterns of atrophy instead of single measures and the former approach has proven to be more sensitive (McEvoy et al. 2011; Westman et al. 2011c; Zhang et al. 2011). MRI is today an integrated part of the suggested research (Dubois et al. 2007) and diagnostic criterion (McKhann et al. 2011) alongside cerebrospinal fluid (CSF) markers and positron emission tomography (PET).

Freesurfer is a highly automated structural MRI image processing pipeline which produces regional volume, cortical thickness, gray matter volume, surface area, mean curvature, gaussian curvature, folding index and curvature index measures. Automated image analysis pipelines may have particular advantages when it comes to widespread uptake in either clinical or research practice. Manual measures of different brain regions are time consuming and operator dependent and therefore not always practical in a clinical settings. However, automated tools must be precise, accurate, fast and must be validated and tested on large cohorts. Several groups have utilized automated pipelines in AD research (Cui et al. 2011; Li et al. 2011; McEvoy et al. 2009, 2011). We have also previously used automated image analysis pipeline output analyzed with multivariate tools for the purpose of AD classification and to predict conversion from the prodromal stage of the disease, mild cognitive impairment (Westman et al., 2011a, b). Different regional MRI measures have been used in the studies reported in the literature including our own and different approaches have been taken to normalization. For example, should regional volumes be normalized by dividing by intracranial volume to reflect differences in head size between individuals, particularly males and females, and pre-morbid brain size? It is not clear yet which combination of regional measures and which normalization approaches yield the best results for individual classification and prediction.

The current study investigated the use of regional MRI measures analyzed by orthogonal partial least square to latent structure (OPLS) a multivariate tool for classification of individual subjects. The specific aims were to determine: (1) which type of normalization approach is most useful for the different regional measures (2) which combination of regional measures results in the best classification accuracy when distinguishing between AD subjects and healthy controls, and (3) to prospectively predict conversion from MCI to AD at baseline by appropriate choice of multivariate model. We hypothesized that regional volumetric measures would give the best results when normalized by total intracranial volume, that surface area should be normalized by whole brain surface area, while the remaining measures (cortical thickness, mean curvature, gaussian curvature, folding index and curvature index) should not be normalized. Further, we hypothesized that a combination of regional subcortical volumes normalized by intracranial volume and un-normalized cortical thickness measures would generate the most accurate predictions.

## Materials and Methods

### Data

Data was downloaded from the Alzheimer’s disease Neuroimaging Initiative (ADNI) database (www.loni.ucla.edu/ADNI, PI Michael M. Weiner). ADNI was launched in 2003 by the National Institute on Aging (NIA), the National Institute of Biomedical Imaging and Bioengineering (NIBIB), the Food and Drug Administration (FDA), private pharmaceutical companies and non-profit organizations, as a $60 million, 5-year public–private partnership. The primary goal of ADNI has been to test whether serial MRI, PET and other biological markers are useful in clinical trials of MCI and early AD. Determination of sensitive and specific markers of very early AD progression is intended to aid researchers and clinicians to develop new treatments and monitor their effectiveness, as well as lessen the time and cost of clinical trials. ADNI subjects aged 55–90 from over 50 sites across the U.S. and Canada participated in the research and more detailed information is available at www.adni-info.org.

### Inclusion and Diagnostic Criteria

A total of 699 subjects were included in the current study (AD = 187, MCI = 287 and CTL = 225). The demographics of the cohort are given in Table 1. We included all subjects who had successful MRI measures at baseline which passed the quality control steps outlined below. Out of the 287 MCI subjects, 87 had converted at the 18-month follow-up (MCIc) to Alzheimer’s disease. Subjects which did not convert to Alzheimer’s disease at 18 month follow up are referred to as MCI stable (MCIs) here.

**Table 1:** Subject characteristics

	AD (*n* = 187)	MCI (*n* = 287)	CTL (*n* = 225)	MCIs (*n* = 200)	MCIc (*n* = 87)	*p*
Female/male	88/99	104/183	108/117	66/134	38/49	–
Age	75.4 ± 7.5	74.9 ± 7.0	75.9 ± 5.1	74.7 ± 7.1	75.2 ± 6.9	–
Education	14.7 ± 3.1	15.8 ± 3.0	16.1 ± 2.9	15.9 ± 3.0	15.4 ± 3.0	–
MMSE	23.3 ± 2.0^a,b^	27.1 ± 1.7^b^	29.1 ± 1.0	27.4 ± 1.7	26.5 ± 1.7	<0.001
CDR	0.7 ± 0.3^a,b^	0.5^b^	0	0.5	0.5	<0.001

Data are represented as mean ± standard deviation

Two-way Student *t* test with Bonferroni correction was used for age and education and neuropsychological tests comparisons. ^a^ Significant compared to MCI group. ^b^ Significant compared to control group

*AD* Alzheimer’s disease, *MCI* mild cognitive impairment, *CTL* healthy control, Education in years, *MMSE* mini mental state examination, *ADAS1* Word list non-learning (mean), *CDR* clinical dementia rating. Chi-square was used for gender comparison

A detailed description of the inclusion criteria can be found on the ADNI webpage (http://www.adni-info.org/Scientists/AboutADNI.aspx#). Subjects were between 55 and 90 years of age. They had a study partner able to provide an independent evaluation of functioning, and spoke either English or Spanish. All subjects were willing and able to undergo all test procedures including neuroimaging and agreed to longitudinal follow up. Specific psychoactive medications were excluded.

Alzheimer’s disease (*General inclusion/exclusion criteria*): (1) Mini mental state examination (MMSE) scores between 20 and 26, (2) Clinical dementia rating scale (CDR) of 0.5 or 1.0, 3) met NINCDS/ADRDA criteria for probable AD, (3) Geriatric Depression Scale <6, (4) Subjects excluded if they had any other significant neurologic disease other than Alzheimer’s disease.

Mild cognitive impairment (*General inclusion/exclusion criteria*): (1) subjects had MMSE scores between 24 and 30 (inclusive), (2) memory complaint, with objective memory loss measured by education adjusted scores on the Wechsler Memory Scale Logical Memory II, (3) CDR of 0.5, (4) absence of significant levels of impairment in other cognitive domains, essentially preserved activities of daily living, and an absence of dementia, (5) Geriatric Depression Scale <6, (6) Subjects excluded if they had any other significant neurologic disease other than Alzheimer’s disease.

Controls (*General inclusion/exclusion criteria*): (1) MMSE scores between 24 and 30 inclusive, (2) CDR of zero, (3) they were non-depressed, non MCI, and non-demented.

### MRI

MRI data was downloaded from the ADNI website (www.loni.ucla.edu/ADNI). A description of the data acquisition for the ADNI study can be found at www.loni.ucla.edu/ADNI/research/Cores/index.shtml. Briefly, 1.5T MRI data was collected from a variety of MR-systems with protocols optimized for each type of scanner. The MRI protocol included a high resolution sagittal 3D T1-weighted MPRAGE volume (voxel size 1.1 × 1.1 × 1.2 mm^3^) acquired using a custom pulse sequence specifically designed for the ADNI study to ensure compatibility across scanners. Full brain and skull coverage was required for the MRI datasets and detailed quality control carried out on all MR images from both studies according to previously published quality control criteria (Simmons et al. 2009, 2011).

### Regional Volume Segmentation and Cortical Thickness Parcellation

**Fig. 1:**
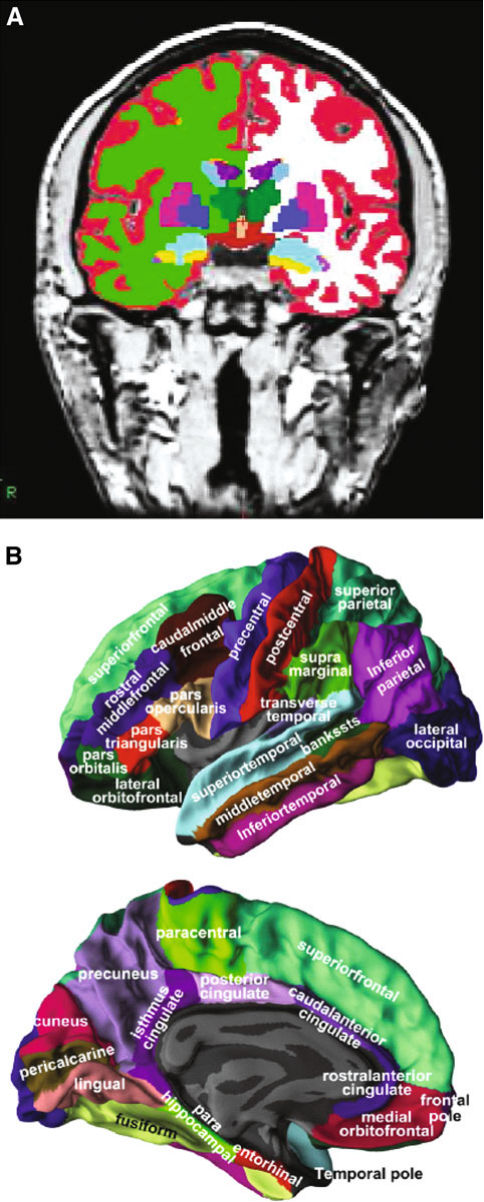
Representations of ROIs included as candidate input variables in the multivariate OPLS model. **a** Coronal view of a T_1_-weighted MPRAGE image displaying the regional volumes. **b** Lateral and medial views of the *grey matter* surface illustrating the 34 regional cortical thickness measures

We utilized the Freesurfer pipeline version 5.1.0 (http://surfer.nmr.mgh.harvard.edu/), which includes removal of non-brain tissue using a hybrid watershed/surface deformation procedure (Segonne et al. 2004), automated Talairach transformation, segmentation of the subcortical white matter and deep grey matter volumetric structures (Fischl et al. 2002; Fischl et al. 2004a; Segonne et al. 2004) intensity normalization (Sled et al. 1998), tessellation of the grey matter white matter boundary, automated topology correction (Fischl et al. 2001; Segonne et al. 2007), and surface deformation following intensity gradients to optimally place the grey/white and grey/cerebrospinal fluid borders at the location where the greatest shift in intensity defines the transition to the other tissue class (Dale et al. 1999; Dale and Sereno 1993; Fischl and Dale 2000). Once the cortical models are complete, registration to a spherical atlas takes place which utilizes individual cortical folding patterns to match cortical geometry across subjects (Fischl et al. 1999). This is followed by parcellation of the cerebral cortex into units based on gyral and sulcal structure (Desikan et al. 2006; Fischl et al. 2004b). The pipeline generated 68 cortical thickness, cortical volume, surface area, mean curvature, gaussian curvature, folding index and curvature index measures (34 from each hemisphere) and 46 regional subcortical volumes. Volumes of white matter hypointensities, optic chiasm, right and left vessel, and left and right choroid plexus were excluded from further analysis. Cortical thickness and volumetric measures from the right and left side were averaged (Fjell et al. 2009; Walhovd et al. 2011). In total 259 variables obtained from the pipeline were used as input variables for the OPLS classification, 34 cortical regions (7 types of measures) and 21 regional volumes (Table 2). Figure 1 illustrates the location of both the cortical and subcortical regions. This segmentation approach has been used for multivariate classification of Alzheimer’s disease and healthy controls (Westman et al. 2011d), neuropsychological-image analysis (Liu et al. 2010c, 2011), imaging-genetic analysis (Liu et al. 2010a, b) and biomarker discovery (Thambisetty et al. 2010).

**Table 2:** Variable included in OPLS analysis

Cortical measures^a^	Subcortical measures^b^
Banks of superior temporal sulcus	Third ventricle
Caudal anterior cingulate	Fourth ventricle
Caudal middle frontal gyrus	Inferior lateral ventricle
Cuneus cortex	Lateral ventricle
Entorhinal cortex	Cerebrospinal fluid (CSF)
Fusiform gyrus	Accumbens
Inferior parietal cortex	Amygdala
Inferior temporal gyrus	Brainstem
Isthmus of cingulate cortex	Caudate
Lateral occipital cortex	Cerebellum cortex
Lateral orbitofronral cortex	Cerebellum white matter
Lingual gyrus	Corpus callosum anterior
Medial orbitalfrontal cortex	Corpus callosum central
Middle temporal gyrus	Corpus callosum midanterior
Parahippocampal gyrus	Corpus callosum midposterior
Paracentral sulcus	Corpus callosum posterior
Frontal operculum	Hippocampus
Orbital operculum	Putamen
Triangular part of inferior frontal gyrus	Pallidum
Pericalcarine cortex	Thalamus proper
Postcentral gyrus	Ventral diencephalon (DC)
Posterior cingulate cortex	
Precentral gyrus	
Precuneus cortex	
Rostral anterior cingulate cortex	
Rostral middle frontal gyrus	
Superior frontal gyrus	
Superior parietal gyrus	
Superior temporal gyrus	
Supramarginal gyrus	
Frontal pole	
Temporal pole	
Transverse temporal cortex	
Insular	

259 variables in total included in OPLS analysis

^a^Cortical measures = 34 regions (cortical volumes, cortical thickness, surface area, mean curvature, gaussian curvature, folding index and curvature index)

^b^Subcortical measures = 21 regions (volumes)

### Normalization

We wished to compare the effect of different normalisation approaches on multivariate analysis to determine which gave the best discriminant and predictive performance. To this end we normalised the various MRI measures in a series of ways. All sets of regional MRI measures from each subject were considered in their raw form and also normalized by the subject’s intracranial volume. Further, the cortical thickness measures and the surface area measures from each subject were also normalized by the subject’s average global cortical thickness and the subject’s total surface area respectively.

### Statistical Analysis

MRI measures were analyzed using OPLS (Bylesjo et al. 2007; Trygg and Wold 2002; Rantalainen et al. 2006; Westman et al. 2011c, 2010; Wiklund et al. 2008), a supervised multivariate data analysis method included in the software package SIMCA (Umetrics AB, Umea, Sweden). A very similar method, partial least squares to latent structures (PLS) has previously been used in several studies to analyze MR-data (Levine et al. 2008; McIntosh and Lobaugh 2004; Oberg et al. 2008; Westman et al. 2009, 2007). OPLS and PLS give the same predictive accuracy, but the advantage of OPLS is that the model created to compare groups is rotated, which means that the information related to class separation is found in the first component of the model, the predictive component. The other orthogonal components in the model, if any, relate to variation in the data not connected to class separation. Focusing the information related to class separation on the first component makes data interpretation easier (Wiklund et al. 2008). There are also many similarities between linear support vector machine (SVM) and OPLS. Both methods can handle datasets with more dimensions than samples. Linear SVM weights illustrate the importance of the variables for the classification in descending order in the same way as the loadings plots do for OPLS. The unique property of OPLS when compared to other linear regression methods is its ability to separate the modeling of correlated variation from structured noise (uncorrelated variation). The structured noise is defined as orthogonal variation in Y. At the same time the model maximizes the covariance between X and Y.

Pre-processing was performed using mean centring and unit variance scaling. Mean centring improves the interpretability of the data, by subtracting the variable average from the data. By doing so the data set is repositioned around the origin. Large variance variables are more likely to be expressed in modeling than low variance variables. Consequently, unit variance scaling was selected to scale the data appropriately. This scaling method calculates the standard deviation of each variable. The inverse standard deviation is used as a scaling weight for each MR-measure.

The results from the OPLS analysis are visualized in a scatter plot by plotting the predictive component, which contains the information related to class separation. Components are vectors, which are linear combinations of partial vectors and are dominated by the input variables (x), in this case the regional MRI output. Each point in the scatter plot represents one individual subject.

Each model receives a Q^2^(Y) value that describes its statistical significance for separating groups. Q^2^(Y) values >0.05 are regarded as statistically significant (Umetrics 2008), where

1 $$ Q^{2} (Y) = 1 - {\text{PRESS/SSY}} $$

where PRESS (predictive residual sum of squares) = Σ(y_actual _− y_predicted_)^2^ and SSY is the total variation of the Y matrix after scaling and mean centring (Eriksson et al. 2006). Q^2^(Y) is the fraction of the total variation of the Ys (expected class values) that can be predicted by a component according to cross validation (CV). CV is a statistical method for validating a predictive model which involves building a number of parallel models. These models differ from each other by leaving out a part of the data set each time. The data omitted is then predicted by the respective model. In this study we used seven fold CV, which means that 1/7th of the data is omitted for each CV round. Data is omitted once and only once.

Variables can be plotted according to their importance for the separation of groups. The plot shows the MRI measures and their corresponding jack-knifed confidence intervals. Jack-knifing is used to estimate the bias and standard error. Measures with confidence intervals that include zero have low reliability (Wiklund et al. 2008). Covariance is plotted on the y-axis, where

2 $$ Cov(t,X_{i} ) = t^{T} X_{i} /(N - 1) $$

where t is the transpose of the score vector t in the OPLS model, i is the centered variable in the data matrix X and N is the number of variables (Wiklund et al. 2008). A measure with high covariance is more likely to have an impact on group separation than a variable with low covariance. MRI measures below zero in the scatter plot have lower values in AD subjects compared to CTL subjects, while MRI measures above zero are higher in AD subjects compared to CTL subjects in the model.

Altogether eight types of regional MRI measures were used (cortical thickness, cortical volumes, subcortical volumes surface area, mean curvature, gaussian curvature, folding index and curvature index), resulting in a total of 259 variables to be used for OPLS analysis (Table 2). A series of OPLS models were created for comparing the CTL versus AD groups. For each of the eight types of measures, both raw measures and measures normalized by intracranial volume (ICV) were used in these models. In addition cortical thickness measures were also normalised by mean cortical thickness and surface area measures by total surface area. Subsequently hierarchical models consisting of combinations of two or three sets of regional measures were also created (for example raw cortical thickness measures and raw subcortical volumes, or cortical thickness measures normalised by intracranial volume and subcortical volumes normalised by intracranial volume). Feature selection was not used other than excluding measures which resulted in non-significant models. Excluding specific regions from the models might make the models less representative and structural features measured from a limited set of pre-defined regions might not be able to reflect the pattern of structural abnormalities in their entirety (Zhang et al. 2011). Further, Cuignet et al. (2011) showed that feature selection does not improve the classification but it does increase the computational time. Another recent paper investigated the effect of feature selection (Chu et al. 2012) and they concluded that feature selection improves the results particularly for small cohorts but it does not seem to have a great affect on larger samples. We have a much larger sample than the largest sample used in this latter study.

The MCI subjects were also assessed against the best CTL versus AD models to investigate how well the model could predict conversion at 18 month follow-up from baseline. Sensitivity, specificity, accuracy and area under the receiver operating characteristic curve (AUC) of the different models were calculated from the cross-validated prediction values of the OPLS models. Areas under the receiver operating characteristic curve were compared by using the method of Hanley and McNeil (1983; McEvoy et al. 2011), *p*-values <0.05 after correcting for multiple comparisons using Bonferroni correction were considered statistically significant.

The two-way Student *t* test with Bonferroni correction (*p*-values > 0.05 considered significant) was used for univariate analysis to investigate the effect of normalization of single regional measures (Tables 6; 7).

## Results

OPLS models were created using CTL versus AD data for all eight types of regional MRI measures (cortical thickness, cortical volume, subcortical volume, surface area, mean curvature, gaussian curvature, folding index and curvature index) for both raw data and normalized data. Hierarchical models were also created using up to three types of different regional MRI measures. No feature selection was performed for any of the eight different types of measures, meaning all data was included.

Modeling and quality parameters are only shown for the statistically significant single measure models and the most robust [highest Q^2^(Y)] hierarchical models (Table 3: raw data, Table 4: ICV normalized data, Table 5: data normalized by total surface area, average cortical thickness and mixed models including both normalized and raw data).

**Table 3:** Raw data (data not normalized)

Q^2^(Y) = predictive ability of model and AUC = area under the curve. Confidence interval within parentheses. Thick line separating models (within the table content) means that the block of models are significantly different in AUC compared to the other models in that category of normalization method and number of input measures

* Significant difference in AUC between raw data versus normalized with intra cranial volume. ** Significant difference in AUC between raw data and normalized with intra cranial volume and raw data versus normalized with mean cortical thickness. *P*-values <0.05 considered significant after Bonferroni correction

**Table 4:** Data normalized by intra cranial volume

Q^2^(Y) = predictive ability of model and AUC = area under the curve. Confidence interval within parentheses. Thick line separating models (within the table content) means that the block of models are significantly different in AUC compared to the other models in that category of normalization method and number of input measures

* Significant difference in AUC between raw data versus normalized with intra cranial volume. *P*-values <0.05 considered significant after Bonferroni correction

**Table 5:** Other normalization models

Model	Q^2^(Y)	Accuracy		Sensitivity		Specificity		AUC
CT (normalized with average CT)	0.476	83.7	(80.0-87.0)	83.0	(76.8-87.6)	84.8	(79.6-88.9)	0.912**
SA (normalized with total SA)	0.169	69.2	(64.6-73.4)	71.1	(64.3-77.4)	67.6	(61.2-73.3)	0.744
**Hierarchial model**								
SV + CT*	0.603	89.3	(86.0-92.0)	87.2	(81.6-91.2)	91.1	(86.7-94.2)	0.951
CV + CT*	0.592	88.4	(84.9-91.1)	86.6	(81.0-90.8)	89.8	(85.1-93.1)	0.951
**Hierarchial model**								
SV + CV + CT*	0.626	91.5	(88.4-93.8)	89.8	(86.5-92.4)	92.9	(88.8-95.6)	0.960

Q^2^(Y) = predictive ability of model, *CV* cortical volume, *SV* subcortical volume, *CT* cortical thickness, *SA* surface area and *AUC* area under the curve. Confidence interval within parentheses. * Mixed model = SV and CV normalized by ICV with raw CT data. ** Significant difference in AUC between raw data and normalized with mean cortical thickness. *P*-values <0.05 considered significant after Bonferroni correction

**Fig. 2:**
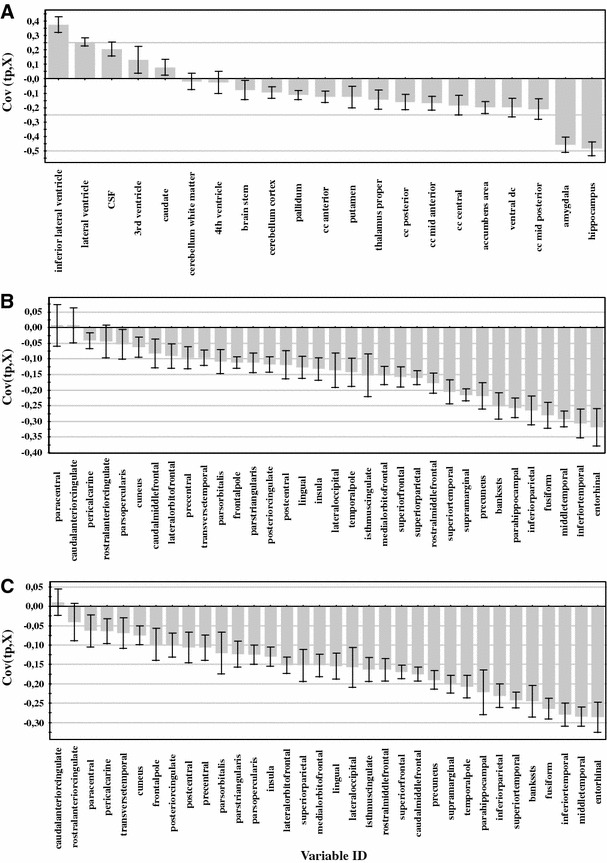
Variables of importance for the separation between CTL versus AD. **a** subcortical volumes (normalized by intra cranial volume) **b** cortical *gray matter* volumes (normalized by intra cranial volume) **c** cortical thickness (raw data). Measures above zero have a larger value in AD compared to controls and measures below zero have a lower value in AD compared to controls. A measure with a high covariance is more likely to have an impact on group separation than a measure with a low covariance. Measures with jack knifed confidence intervals that include zero have low reliability

Figure 2 shows the variables of importance for the three most robust single measure models [subcortical volumes (ICV normalized), cortical volumes (ICV normalized) and cortical thickness measures (raw data)]. Variables of greatest importance for the separation between groups were as expected the medial temporal lobe structures such as hippocampus, amygdala and entorhinal cortex. To illustrate the effect of normalization approaches on single measures univariate analysis was performed for subcortical volumes (Table 6), cortical volumes and cortical thickness measures (Table 7). As can be observed in Tables 6 and 7, normalizing volumes with ICV and raw cortical thickness measures gave the best results.

**Table 6:** Univariate analysis of subcortical volumes using different normalization approaches for AD versus CTL

Subcortical measures	Subcortical volumes	
Normalization	Raw	ICV
Third ventricle	ns	***p*** ** < 0.01**
Fourth ventricle	ns	ns
Inferior lateral ventricle	*p* < 0.00001	*p* < 0.00001
Lateral ventricle	*p* < 0.00001	*p *< 0.00001
Cerebrospinal fluid (CSF)	*P* < 0.001	*p* < 0.00001
Accumbens	*p* < 0.00001	*p* < 0.00001
Amygdala	*p* < 0.00001	*p* < 0.00001
Brainstem	ns	***p*** **< 0.00001**
Caudate	ns	ns
Cerebellum cortex	ns	ns
Cerebellum white matter	ns	ns
Corpus callosum anterior	ns	ns
Corpus callosum central	*p* < 0.001	*p* < 0.001
Corpus callosum midanterior	*p* < 0.01	***p*** ** < 0.001**
Corpus callosum midposterior	*p* < 0.001	***p*** ** < 0.00001**
Corpus callosum posterior	ns	***p*** ** < 0.01**
Hippocampus	*p* < 0.00001	*p* < 0.00001
Putamen	ns	ns
Pallidum	ns	ns
Thalamus proper	ns	ns
Ventral diencephalon (DC)	*p* < 0.001	***p*** ** < 0.00001**

Bold values illustrate that there are differences in normalization method

AD = Alzheimer’s disease, CTL = healthy control, ICV = intra cranial volume and raw = not normalized. Two-way Student *t* test with Bonferroni correction is used for univariate analysis. *P*-values <0.05 considered significant after Bonferroni correction

**Table 7:** Univariate analysis of cortical thickness and volumes using different normalization approaches for AD versus CTL

Cortical measures	Cortical thickness			Cortical volume	
Normalization	Raw	ICV	Mean CT	Raw	ICV
Banks of superior temporal sulcus	*p* < 0.00001	*p* < 0.00001	*p* < 0.00001	*p* < 0.00001	*p* < 0.00001
Caudal anterior cingulate	ns	ns	*p* < 0.00001	Ns	ns
Caudal middle frontal gyrus	***p*** ** < 0.00001**	*p* < 0.001	ns	Ns	ns
Cuneus cortex	ns	ns	*p* < 0.00001	Ns	ns
Entorhinal cortex	*p* < 0.00001	*p* < 0.00001	*p* < 0.00001	*p* < 0.00001	*p* < 0.00001
Fusiform gyrus	*p* < 0.00001	*p* < 0.00001	*p* < 0.00001	*p* < 0.00001	*p* < 0.00001
Inferior parietal cortex	*p* < 0.00001	*p* < 0.00001	*p* < 0.00001	*p* < 0.00001	*p* < 0.00001
Inferior temporal gyrus	*p* < 0.00001	*p* < 0.00001	*p* < 0.00001	*p* < 0.00001	*p* < 0.00001
Isthmus of cingulate cortex	***p*** ** < 0.00001**	*p* < 0.001	ns	*p* < 0.001	***p*** ** < 0.00001**
Lateral occipital cortex	***p*** ** < 0.00001**	*p* < 0.05	ns	Ns	***p*** ** < 0.001**
Lateral orbitofronral cortex	***p*** ** < 0.00001**	ns	ns	Ns	***p*** ** < 0.05**
Lingual gyrus	***p*** ** < 0.00001**	ns	*p* < 0.01	*p* < 0.05	***p*** ** < 0.01**
Medial orbitalfrontal cortex	***p*** ** < 0.00001**	*p* < 0.01	Ns	*p* < 0.01	***p*** ** < 0.00001**
Middle temporal gyrus	*p* < 0.00001	*p* < 0.00001	*p* < 0.00001	*p* < 0.00001	*p* < 0.00001
Parahippocampal gyrus	*p* < 0.00001	*p* < 0.00001	*p* < 0.00001	*p* < 0.00001	*p* < 0.00001
Paracentral sulcus	ns	ns	*p* < 0.00001	Ns	ns
Frontal operculum	*p* < 0.00001	ns	*p* < 0.00001	Ns	ns
Orbital operculum	***p*** ** < 0.00001**	ns	ns	*p* < 0.05	***p*** ** < 0.01**
Triangular part of inferior frontal gyrus	***p*** ** < 0.00001**	ns	*p* < 0.01	*p* < 0.01	***p*** ** < 0.001**
Pericalcarine cortex	ns	ns	*p* < 0.00001		
Postcentral gyrus	***p*** ** < 0.01**	ns	*p* < 0.00001		***p*** ** < 0.01**
Posterior cingulate cortex	***p*** ** < 0.001**	ns	*p* < 0.01	*p* < 0.01	***p*** ** < 0.00001**
Precentral gyrus	***p*** ** < 0.001**	ns			
Precuneus cortex	***p*** ** < 0.00001**	*p* < 0.00001		*p* < 0.00001	*p* < 0.00001
Rostral anterior cingulate cortex	ns	ns	*p* < 0.01	Ns	ns
Rostral middle frontal gyrus	***p*** ** < 0.00001**	*p* < 0.001	ns	*p* < 0.001	***p*** ** < 0.00001**
Superior frontal gyrus	***p*** ** < 0.00001**	*p* < 0.01	ns	*p* < 0.01	***p*** ** < 0.00001**
Superior parietal gyrus	***p*** ** < 0.00001**	*p* < 0.01	ns	*p* < 0.01	***p*** ** < 0.00001**
Superior temporal gyrus	***p*** ** < 0.00001**	*p* < 0.00001	ns	*p* < 0.00001	*p* < 0.00001
Supramarginal gyrus	***p*** ** < 0.00001**	*p* < 0.00001	ns	*p* < 0.00001	*p* < 0.00001
Frontal pole	***p*** ** < 0.001**	ns	ns	*p* < 0.001	*p* < 0.001
Temporal pole	***p*** ** < 0.00001**	*p* < 0.00001	ns	*p* < 0.00001	*p* < 0.00001
Transverse temporal cortex	ns	ns	ns	Ns	***p*** ** < 0.01**
Insular	***p*** ** < 0.00001**	ns	ns	Ns	***p*** ** < 0.0001**

Bold values illustrate that there are differences in normalization method

AD = Alzheimer’s disease, CTL = healthy control, ICV = intra cranial volume, Raw = not normalized and mean CT = normalized by mean cortical thickness. Two-way Student *t* test with Bonferroni correction is used for univariate analysis

### AD Classification and MCI Conversion

**Fig. 3:**
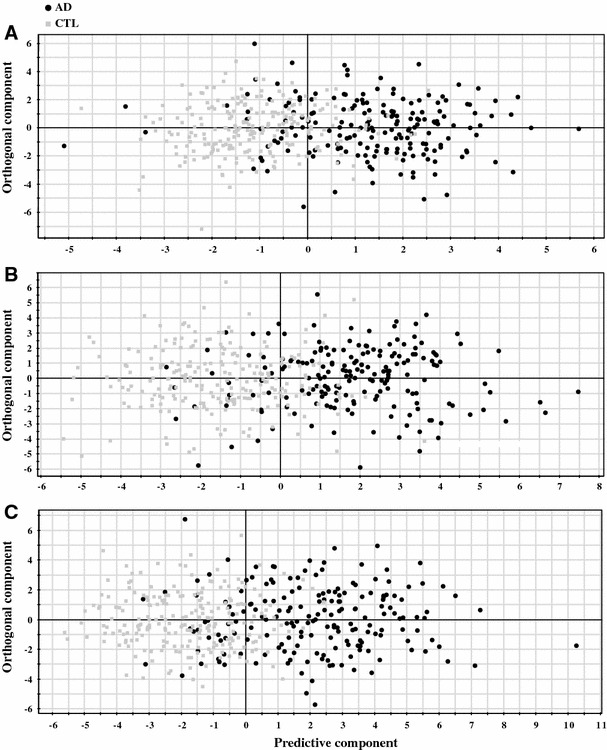
Scatter plots illustrating the separation between CTL versus AD. **a** subcortical volumes (normalized by intra cranial volume) **b** cortical gray matter volumes (normalized by intra cranial volume) **c** cortical thickness (raw data). The *scatter plots* visualise group separation and the predictability of three different AD versus CTL models. Each *black circle* represents an AD subject and each *gray square* a control subject. Control subjects to the *right* of zero and AD subjects to the *left* of zero are falsely predicted

The results from the different models used for AD classification can be observed in Tables 3, 4, 5. The AUC values for the different models were compared using the method of Hanley and McNeil and *p*-values <0.05 after Bonferroni correction for multiple comparisons were considered statistically significant. Only five out of the eight single measure models gave significant results for both raw and normalized data. Gaussian curvature, folding index and curvature index were therefore excluded from further analysis. Out of the five remaining measures the best results were obtained from the cortical thickness, cortical volume and subcortical volume measures (these measures were significantly different from surface area and mean curvature for both raw data and data normalized to ICV). Using raw data the best discrimination was obtained for cortical thickness measures with an accuracy of 85.2 % (significantly different from cortical and subcortical volumes) and for data normalized by ICV the best discrimination was obtained from the cortical volumes with an accuracy of 83.5 % (significantly different from cortical thickness and subcortical volumes). Looking at the normalization approaches for the single measures, a significantly better result was obtained for the cortical thickness measures when the data was not normalized, compared to normalization with ICV and mean cortical thickness (85.2 % compared to 83.5 and 83.7 % respectively). For cortical volumes it was significantly better to normalize with ICV than to use raw data (83.5 % compared to 81.8 %). For the other measures normalization did not have an effect. Figure 3 shows the scatter plots for the top three single measures (raw data cortical thickness and ICV normalized cortical and subcortical volumes) illustrating the separation between the AD and CTL groups.

For the hierarchical models containing a combination of two different measures the best models were the combination of subcortical volumes with either cortical thickness or cortical volumes for both raw data and ICV normalization (Raw data: subcortical volumes + cortical thickness = 89.8 %, subcortical volumes + cortical volumes = 88.6 %. ICV normalized data: subcortical volumes + cortical volumes = 89.8 %, subcortical volumes + cortical thickness = 88.4 %). There seemed to be no effect of normalization approaches for the most accurate and robust models combining two different measures. No effect of normalization approach was observed either for the models containing three different measures.

Combining three different measures did not significantly affect the prediction accuracy compared to using two different measures. Only the three best models are shown using the combination of cortical thickness, cortical volume and subcortical volumes. However, the best overall prediction accuracy was obtained using this combination with raw cortical thickness data and volumes normalized to ICV (91.5 %).

Finally the best AD versus CTL models containing the measures cortical thickness, cortical volumes and subcortical volumes were used to predict conversion at 18 month follow-up. Out of 287 MCI subjects 87 had converted to AD at follow up. Similar results were observed for the MCI predictions as for the models discriminating between AD patients and cognitively normal subjects (Table 8). The best results were obtained with a hierarchical model of two sets of measures when subcortical volumes were combined with either cortical volumes or cortical thickness. Combining the three measures did not improve the predictions and normalization approach did not seem to significantly affect the results either. The best results were obtained from the two models combining cortical thickness with subcortical volumes (both ICV normalized data and mixed data where the volumes are normalized to ICV and the raw cortical thickness data was used) with 77 % of the MCIc subjects correctly classified.

**Table 8:** MCI predictions using the CTL versus AD models as training data

Raw data	Sensitivity (%)	Specificity (%)	Accuracy (%)	AUC
SV + CV	75.9	60.5	65.1	0.734
SV + CT	74.7	62.5	66.2	0.746
CV + CT	67.8	69.0	68.6	0.742
SV + CV + CT	75.9	64.0	67.6	0.753
Average	**73.6**	**64.0**	**66.9**	**0.744**
ICV normalized				
SV + CT	77.0	65.0	68.6	0.739
SV + CV	75.9	60.5	65.1	0.729
CV + CT	68.9	67.0	67.6	0.736
SV + CV + CT	73.5	64.0	66.9	0.743
Average	**73.8**	**64.1**	**67.1**	**0.737**
Mixed models*				
SV + CT	77.0	64.5	68.3	0.749
CV + CT	70.1	67.0	67.9	0.743
SV + CV + CT	75.9	66.5	69.3	0.748
Average	**74.3**	**66.0**	**68.5**	**0.747**

*CV* cortical volume, *SV* subcortical volume, *CT* cortical thickness, *AD* Alzheimer’s disease, *CTL* healthy control, *MCI* mild cognitive impairment and *ICV* intra cranial volume. 287 MCI subjects are predicted on to the different AD versus CTL models, 87 MCI converters and 200 MCI stable. Sensitivity = MCI converters predicted as AD and specificity = MCI stable predicted as CTL. * Mixed model = SV and CV normalized by ICV and raw CT data

## Discussion

The Freesurfer pipeline has been utilized in a number of studies for AD classification and predicting MCI conversion (Cui et al. 2011; Cuingnet et al. 2011; McEvoy et al. 2009, 2011; Westman et al. 2011a, b), but the complete range of measures which can be obtained have not yet been fully explored.

### Normalization

The way in which different regional measures such as volumes and cortical thickness should be normalized is very important. Previous studies (Barnes et al. 2010; Cui et al. 2011; Farias et al. 2011; Fjell et al. 2009; Walhovd et al. 2011; Westman et al. 2011b) have utilized different approaches which can make results difficult to compare. The results from the present study indicate that cortical thickness measures should not be normalized, while volumes should probably be normalized to ICV. Normalizing cortical volumes improved the classification accuracy while normalizing subcortical volumes did not show any statistically significant improvement using the single measure OPLS multivariate models. Further, looking at the single regions (Tables 6, 7), it seems that normalizing the volumes results in the largest differences while using the raw data for cortical thickness yields the best results. When combining the different measures in multivariate models the normalization effect disappears. A potential explanation for this could be that the use of multiple regional measures provides enough anatomical information about the brain atrophy pattern such that multivariate models are robust enough to handle the variation caused by different normalization approaches. In a recent paper, the consistency of volumetric measures derived by FreeSurfer was investigated in five different cohorts (a total of 883 subjects) (Walhovd et al. 2011). This study normalized regional volume measurements by ICV as we propose here. They concluded that ICV normalization is the most commonly used normalization approach in the literature. However, it has also been stated that normalizing to ICV is unlikely to be adequate due to the non-linear relationship between volumes and ICV which was found in a sample of 78 healthy controls (Barnes et al. 2010). Another recent study also stated that normalizing volumes to ICV is inadequate due to the fact that the maximal brain size seems to be an important predictor of cognition in old age, independent of brain pathology (Farias et al. 2011). By normalizing to ICV, the authors claim that investigators may overlook the effect of ICV itself. Especially in longitudinal studies, ICV may be an important variable in itself for quantifying the effect of brain reserve (Farias et al. 2011). Reviewing the literature regarding normalizing cortical thickness, there seems to be a common agreement that these measures should not be normalized (Fjell et al. 2009). This is also confirmed in the present study, where normalized cortical thickness measures gave significantly lower prediction accuracies regardless of normalization approach (mean thickness or ICV).

Previous studies have drawn different conclusions on the best normalization approach to adopt for regional MRI measures. However, we feel fairly confident to say from the results of the present study and results from previous studies that cortical thickness should not be normalized. Normalizing volumes seems to be a more complicated issue however. We believe that considering single regions, the best approach is to normalize to ICV. Even though the relationship between ICV and volumes may not be linear and the effect of ICV itself may be removed if data is normalized, we still believe this may be the best approach to take. This is due to the fact that changes in neurodegenerative disorders are relatively small and could be overlooked if data is not normalized. When we consider multivariate models containing multiple brain regions the normalization approach does not seem to be that important. This need to be further validated in larger studies.

### AD Classification and MCI Conversion

Previous studies have utilized different types of regional MRI measures for AD classification and to predict MCI conversion (Cui et al. 2011; Cuingnet et al. 2011; McEvoy et al. 2009, 2011; Westman et al. 2011a, b). Whole brain volume, regional volumes and cortical thickness measures (volumes normalized to ICV and raw cortical thickness data) were included in a recent study (McEvoy et al. 2011). This study obtained a prediction accuracy of 90.4 % between AD and CTL using a smaller sample from the same cohort as in this study (ADNI). This is very similar to the best accuracies obtain in this study ranging between 89.3 and 91.5 %. Data from the ADNI cohort was also utilized in another study, which included cortical thickness measures and regional cortical and subcortical volumes (data not normalized), to train a SVM classifier using AD and CTL data (Cui et al. 2011). The MCI subjects were then used as a test set (in the same way as in the present study) to predict future conversion at baseline. Using the measures mentioned above 57.1 % of the MCIc and 65.5 % of MCIs were correctly classified, compared to 77 and 65.0 % respectively in the present study.

Other recent studies which did not use Freesurfer as input for multivariate analysis have also found results in line with ours. SVM has been successfully utilized with voxel based input with accuracies up to 90.8 % (Chu et al. 2012; Liu et al. 2012). Our results are also in line with those of Zhang et al. (2011) who found a classification accuracy of 93.2 % when combining ROI based MRI measures with FDG-PET and CSF.

## Conclusion

Automated MRI image analysis pipelines can be used as input for multivariate data analysis and machine learning techniques, but there is also the option of using raw images as the input to similar multivariate or machine learning approaches. One of the major advantages of automated analysis pipelines is their use of a number of predefined regions which are easy to interpret and have a defined biological meaning. These have greater face validity as a biomarker of disease than a complex pattern of individual voxels across the brain (McEvoy et al. 2011). This study demonstrates that combining raw cortical thickness measures with subcortical volumes normalized by intracranial volume gives the best prediction accuracy for separating AD subjects from cognitively normal subjects. Adding further measures did not significantly improve the classification accuracy, most likely because these additional measures are also derived from the same regions as the cortical thickness measures and provide similar information. Further, normalization approach does not seem to have such a great effect as we initially hypothesized. We do however believe that volumes should be normalized by ICV and that raw cortical thickness data should be used, especially when looking at single regions or measures. This need to be further validated in alternative cohorts. Finally, the combination of cortical thickness measures with subcortical volumes shows potential for prospectively predicting future conversion to AD from baseline. We believe this is a sensible approach using MRI patterns as a biomarker of disease. Combining this approach with other biomarkers such as CSF markers (Westman et al. 2012) and PET markers is likely to further improve AD classification and MCI conversion accuracy. This will hopefully lead to improved tools to aid AD diagnosis and allow targeting of the right populations for clinical trials.
